# P181: Confirming nosocomial legionella pneumophila serogroup 1 infection by sequence-based typing (SBT)

**DOI:** 10.1186/2047-2994-2-S1-P181

**Published:** 2013-06-20

**Authors:** M Schousboe, DJ Harte, R Podmore

**Affiliations:** 1 Microbiology/Infection Control, Canterbury Health Laboratories, CDHB, Christchurch, New Zealand; 2 Legionella Reference Laboratory, ESR, Wellington, New Zealand; 3 Microbiology, Canterbury Health Laboratories, CDHB, Christchurch, New Zealand

## Introduction

Sequence-based typing (SBT) is a discriminatory method to genotype *Legionella pneumophila* serogroup strains.

## Objectives

Use of SBT to prove correlation between patients’ and environmental isolates was questioned.

## Methods

Regular surveillance cultures for Legionella contamination of a tertiary level hospital’s domestic hot water supply since 1990 recorded isolation of *Legionella pneumophila* serogroup from the water. The hospital received its domestic hot and cold water from its 90 meter deep artesian water bore. An increase in number of Legionella pneumophila isolates from water samples and swabs from showerheads were recorded after reduction in the reticulating water temperature from 60^0^ to 55^0^C in 1998. Stored Legionella isolates from 3 patients suspected of possible nosocomial infections between 1999 and 2006 were tested by Legionella Reference Laboratory, ESR, New Zealand by SBT and results compared with stored environmental isolates from 1999 and 2006. The clinical details of the 3 patients were obtained from patient records and previously summarised reports of the patients’ nosocomial infection histories.

## Results

Legionella isolates from three patients were tested by SBT; two were adult males, identified in 1999, and 2003 and the third, a child, identified in 2006. The isolates were from sputum, tracheal aspirates and, the 2006 isolates, from two bronchial alveolar samples taken with 10 days interval. The environmental isolates were from two shower swabs from different wards than those related to the patients, a sample taken from a water cooler storage tank and one taken from the tap water passing through a water filter.

All the environmental isolates from the hospital belonged to the same unique SBT allele profile 7, 6, 17, 3, 13, 11. The two patients diagnose in 1999 and 2006 had the same SBT allele profile as the hospital water, while the isolates from the patient identified in 2006 had two totally different profiles.

## Conclusion

SBT allele profiles is a useful tool for confirming the relationship between an environmental source and Legionella pneumophila serogroup 1 isolates from a patient with potential nosocomial infection if the environmental isolates has got an unique allele profile.

## Disclosure of interest

None declared

